# Racial Discrimination during Adolescence Predicts Mental Health Deterioration in Adulthood: Gender Differences among Blacks

**DOI:** 10.3389/fpubh.2017.00104

**Published:** 2017-05-29

**Authors:** Shervin Assari, Ehsan Moazen-Zadeh, Cleopatra Howard Caldwell, Marc A. Zimmerman

**Affiliations:** ^1^Department of Psychiatry, University of Michigan, Ann Arbor, MI, USA; ^2^Center for Research on Ethnicity, Culture and Health, School of Public Health, University of Michigan, Ann Arbor, MI, USA; ^3^Psychiatry and Psychology Research Center, Tehran University of Medical Science, Tehran, Iran; ^4^Psychiatric Research Center, Roozbeh Hospital, Tehran University of Medical Sciences, Tehran, Iran; ^5^Department of Health Behavior and Health Education, School of Public Health, University of Michigan, Ann Arbor, MI, USA; ^6^Prevention Research Center, School of Public Health, University of Michigan, Ann Arbor, MI, USA

**Keywords:** Blacks, African-Americans, gender, racial discrimination, anxiety, depression

## Abstract

**Background:**

Despite the existing knowledge regarding the negative mental health consequences of perceived racial discrimination, very few researchers have used a longitudinal design with long-term follow-up periods to explore gender differences in this association over time.

**Objective:**

The current longitudinal study aimed to investigate gender differences in predictive role of an increase in perceived racial discrimination during adolescence for mental health deterioration a decade later when they are transitioning to young adulthood.

**Methods:**

Current study followed 681 Black youths for 18 years from 1994 (mean age 15) to 2012 (mean age 32). All participants spent their adolescence and transition to young adulthood in an economically disadvantaged urban area in the Midwest of the United States. Independent variable was perceived racial discrimination measured in 1999 and 2002. Outcomes were psychological symptoms (anxiety and depression) measured in 1999 and at end of follow-up (2012). Covariates included sociodemographics (age, family structure, and parental employment) measured in 1994. Gender was used to define groups in a multigroup structural equation model to test moderating effects.

**Results:**

Multigroup structural equation modeling showed that among male Black youth, an increase in perceived racial discrimination from age 20 to 23 was predictive for an increase in symptoms of anxiety and depression from age 20 to 32. Among female Black youth, change in perceived racial discrimination did not predict future change in depressive or anxiety symptoms.

**Conclusion:**

While racial discrimination is associated with negative mental health consequences for both genders, male and female Black youth differ in regard to long-term effects of an increase in perceived discrimination on deterioration of psychological symptoms. Black males seem to be more susceptible than Black females to the psychological effects of an increase in racial discrimination over time.

## Introduction

Discrimination, defined as unfair treatment of different categories of people, is a life stressor with significant adverse effects on both physical (1–4) and mental (5–10) health. Racial discrimination is a particular type of discrimination targeting members of racial and ethnic minorities such as Blacks (3, 9, 11–17). Blacks report higher levels of racial discrimination than other minority groups such as Hispanics (7, 18).

Researchers have found that discrimination in general, and racial discrimination in particular, deteriorates mental health of individuals (19–21). It is proposed that discrimination may contribute to mental health problems in a unique way compared to general and non-specific stressors (3, 10, 11, 18, 22, 23). This argument is mainly based on the observations that experiences and perceptions of racial discrimination better predict psychological distress compared with other types of stressors (10, 24). In fact, discrimination influences several dimensions of mental health (18). More interestingly higher levels of discrimination may predict long-term adverse outcomes for mental health, while the converse may not be true (11). These findings suggest that racial discrimination may be a distinct contributor to the development of psychopathology in racial minority groups (18).

Racial discrimination may contribute to mental health problems through several potential mechanisms including: (1) heightened negative psychological stress response, (2) increased physiological stress response, (3) hypervigilance, and (4) increased participation in unhealthy behaviors (3, 25, 26). Racial discrimination is also associated with shorter telomere length, which is a marker of aging (27). Racial discrimination is associated with higher levels of nervousness and anger and higher likelihood of evaluating social interactions as harassing (28). Himmelstein et al. (25) showed that vigilance coping strategies mediate the relationship between perceived discrimination and distress. In a study which included Blacks and Latinos, Brondolo et al. (18) found an association between perceived discrimination and state and trait negative affect even after controlling for other characteristics such as hostility and socioeconomic status.

Gender may alter harmful effects of perceived discrimination (29, 30). Among middle class Black adolescents, perceived racial discrimination better predicted substance use in males than females (30). In another study on a population of Latinos and Blacks, recent discrimination was associated with more smoking among Black men but not among women or Hispanics (31).

Using a longitudinal design, we conducted the current study to investigate gender differences in predictive role of change in perceived (everyday) racial discrimination during adolescence on mental health deterioration, measured by symptoms of anxiety and depression, in Black youth transitioning to young adulthood. Based on higher rates of discrimination against Black men (32), which is consistent with less employment opportunities for Black men (33), we expected worse effect of discrimination on mental health for Black males than Black females.

## Materials and Methods

### Design and Setting

With a longitudinal design, this study was based on the Flint Adolescent Study (FAS), a long-term prospective cohort study of urban youth in Flint, MI, USA.

### Ethics

The study protocol was approved by the University of Michigan Institutional Review Board. All participants signed informed consent or assent forms before each interview. Participants were compensated for their participation in the study.

### Current Analysis

Data for the current analysis came from Waves 1 (year 1995) and 12 (year 2012) when participants were on average 15- and 32-year old, respectively.

### FAS Design and Methods

The FAS was originally designed to follow up a susceptible group of youth at high risk of school dropout and substance use. Participants were interviewed from 1994 to 1997 (average ages 14–17 years old), 1999–2003 (average ages 19–22 years old), and from 2008 to 2012 (average ages 27–29 years old). The age range for youth over the 12 waves of data collection (from 1994 to 2012) was 14–30 years old.

From years 1994 to 1997, structured, face-to-face 50–60 min interviews were conducted with students. From years 2003 to 2008, interviews were conducted in a community setting or by telephone. Participants completed a paper-and-pencil questionnaire about alcohol and substance use, sexual behavior, and other sensitive information after the interview. Respondents were informed that all information was confidential and *sub poena* protected. Interviewers were trained community members and college students, most of whom were native to the area. Analyses on a broad range of variables from the larger study showed no effects by interviewer race or gender (34). At the request of the participating schools, we used passive consent for parents and written assent for participating students. The study had a low refusal rate (*n* = 9) and represented 92% of eligible youth enrolled in the public high schools. Additional study details are reported elsewhere (34).

### FAS Participants and Sampling

Most of the participants were enrolled from four local public high schools, using non-random sampling. The study enrolled participants from the ninth grade (average age 14.5) and followed them through their transition into adulthood. The study enrolled students in the fall semester of ninth grade if they had a grade point average of 3.0 or lower in eighth grade and if they did not have a diagnosis of a developmental disability or emotional impairment. Most participants came from working-class households; however, only in 25% of the families biological parents were married.

### Analytical Sample

Although the original study included Blacks (80%) and Whites (17%), the current study only included male and female Black youth, and it was focused on mental health consequences of perceived racial discrimination. So all participants identifying as White, mixed African-American, and other races (*N* = 169 or 20%) were excluded. In addition, the sample was limited to the participants for whom discrimination and anxiety and depression measures were available. In addition, as the study was interested in the long-term mental health effects of discrimination, we limited the sample to those who were still under follow-up and had provided information regarding their mental health status in 2012. The analytical sample in this study was 681 male and female Black youth. The retention rate was 90% from Waves 1 to 4; 75% from Waves 4 to 8.

### Process

Data were collected during structured face-to-face interviews followed by a brief paper-and-pencil questionnaire for sensitive items conducted either at school or an alternative community location. This study followed students who remained in school, as well as those who dropped out of school. Each interview lasted about 60 min on average. All measures in this study were collected in interview format except the discrimination items that were collected at the end of the interview in the paper-and-pencil questionnaire.

#### Perceived Racial Discrimination

Daily Life-Experiences subscale (35) was used to measure perceived racial discrimination. This measure includes 20 items and asks respondents whether they had experienced racism-related life events or microstressors (36) in the past year and how much each event has bothered them. Some of the items were as follows: “Being ignored, overlooked, or not given service (in a restaurant, store, etc.),” “Your ideas or opinions being minimized, ignored, or devalued,” and “Not being hired for a job.” Response scale ranged from *never happened to me* (0) to *bothers me extremely* (5). A total score was calculated based on the mean of all items, ranging between 0 and 5, with a higher score representing more perceived racial discrimination.

#### Symptoms of Anxiety

Symptoms of anxiety were measured by the brief symptom inventory (37). Six items assessed the frequency of feeling uncomfortable due to symptoms of anxiety during the past week. Response options were on a Likert scale that ranged from 1 (not at all uncomfortable) to 5 (extremely uncomfortable). Items were averaged to form a scale. This scale has high internal consistency and test–retest reliability (38–40) (Cronbach’s alpha = 0.78 at Wave 1).

#### Symptoms of Depression

Depressive symptoms were measured using six items of the brief symptom inventory (37). These items assess the frequency of feeling uncomfortable during the past week due to symptoms of depression. Some of the sample items were feeling hopeless about the future and having no interest in things. Response options on the Likert scale ranged from 1 (not at all uncomfortable) to 5 (extremely uncomfortable). These six items were averaged to form the final scale. This scale has high internal consistency and test–retest reliability and is valid for use with adolescents (39, 40) (Cronbach’s alpha = 0.79 at Wave 1).

#### Covariates

Baseline age, family structure (i.e., intact vs. not intact family), and family socioeconomic status (number of parents who were employed) were used as control variables.

### Data Analysis

We used SPSS 20.0 (IBM Corp.) and AMOS 18.0 for data analysis. Descriptive statistics were reported using frequency tables, as well as mean and SDs. Pearson’s correlation was used to measure bivariate associations at baseline. Paired sample *t* test was used to test change in depressive symptoms and depression over time.

Multigroup structural equation modeling (SEM) was used for multivariable analysis (41, 42). Different models were used for symptoms of anxiety and depression as the main outcomes. AMOS uses a procedure known as full information maximum likelihood in the presence of missing data.

In the first step, we ran the measurement models in the pooled sample as well as each gender that showed comparably good fit in all cases. This allowed us to test our multigroup structural path models where groups were defined based on gender. In our models, age and SES (living in an intact family and parental employment) were covariates. Arrows were drawn from covariates to baseline depressive symptoms, baseline discrimination, and change in depressive symptoms.

Then models with and without constraining of paths across the groups were fitted. Models with and without correlated errors (error variances for corresponding pretest and posttest measures) were also tested. For our final model, we released constraints as we did not gain improvement in goodness of fit in models with constrains. The path coefficients were compared between the groups for statistically significant differences.

Fit statistics included chi-square, the comparative fit index (CFI) (>0.90), the root mean squared error of approximation (RMSEA) (<0.06), and chi-square to degrees of freedom ratio (43–45). Unstandardized and standardized regression coefficients were reported.

## Results

From our total sample of 681 Black youth, 335 were males and 346 were females.

**Figure 1:**
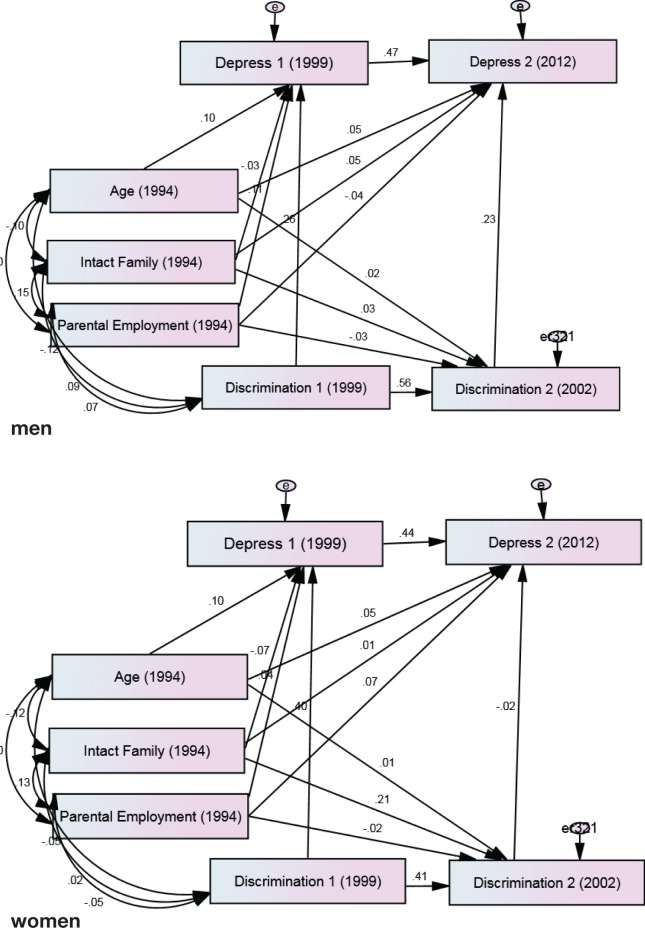
**Summary of path analysis among male and female African-American youth**. Chi-square = 12.591, degrees of freedom = 11, probability level = 0.321, CMIN/DF = 1.145, comparative fit index = 0.991, root mean squared error of approximation = 0.015, and 95% CI = 0.000–0.044.

Our first multigroup SEM on the association between perceived discrimination and depressive symptoms showed a very good fit (chi-square = 12.591, degrees of freedom = 11, probability level = 0.321, CMIN/DF = 1.145, CFI = 0.991, RMSEA = 0.015, and 95% CI = 0.00–0.04). According to this model, change in perceived racial discrimination from 2002 to 2003 predicted change in depressive symptoms from 2000 to 2012 in males (*B* = 0.23, *p* = 0.013) but not females (*B* = −0.02, *p* = 0.857). While gender differences were not found in the effects of age and parental employment, living in an intact family was predictive of change in depressive symptoms in females (*B* = 0.21, *p* = 0.002) but not males (*B* = 0.04, *p* = 0.635) (Figure 1; Table 1).

**Table 1:** **Summary of path analysis between changes in racial discrimination and changes in depressive symptoms among male and female African-American youth**.

	Men		Women	
	*B* (SE)	*P*	*B* (SE)	*P*
**Paths of interest**				
Racial discrimination 2003 → depressive symptoms 2012	0.23 (0.00)	0.013	−0.02 (0.00)	0.857
Racial discrimination 2000 → depressive symptoms 2000	0.26 (0.00)	<0.001	0.40 (0.00)	<0.001
**Auto-regressive paths**				
Racial discrimination 2000 → racial discrimination 2003	0.56 (0.07)	<0.001	0.42 (0.05)	<0.001
Depressive symptoms 2000 → depressive symptoms 2012	0.47 (0.07)	<0.001	0.44 (0.07)	<0.001
**Control paths**				
Age → depressive symptoms 2000	0.10 (0.01)	<0.001	0.10 (0.00)	<0.001
Age → depressive symptoms 2012	0.05 (0.01)	<0.001	0.05 (0.01)	<0.001
Age → racial discrimination 2003	0.02 (0.51)	0.001	0.01 (0.34)	0.002
Two parents working → depressive symptoms 2000	−0.11 (0.10)	0.119	−0.04 (0.09)	0.475
Two parents working → depressive symptoms 2012	−0.05 (0.09)	0.572	0.07 (0.10)	0.344
Two parents working → racial discrimination 2003	−0.03 (9.62)	0.665	−0.02 (6.54)	0.736
Intact family → depressive symptoms 2000	−0.03 (0.10)	0.686	−0.07 (0.10)	0.232
Intact family → depressive symptoms 2012	0.05 (0.10)	0.546	0.02 (0.13)	0.842
Intact family → racial discrimination 2003	0.04 (10.53)	0.635	0.21 (7.84)	0.002

**Figure 2:**
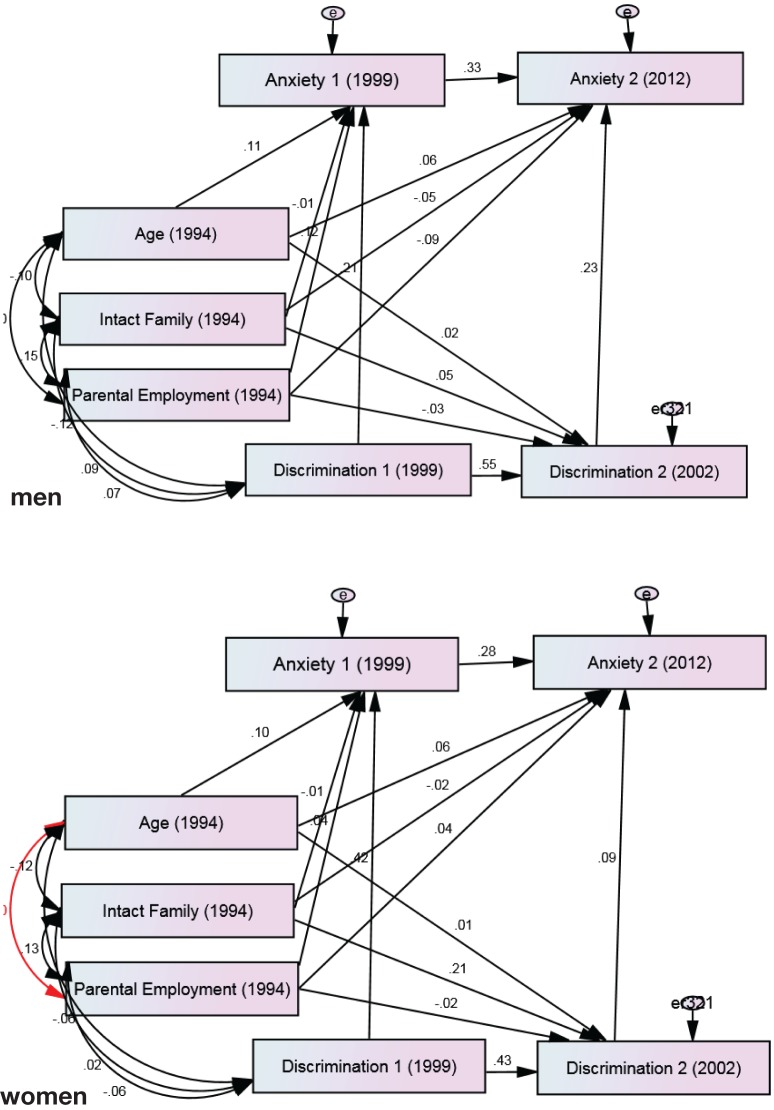
**Summary of path analysis among male and female African-American youth**. Chi-square = 7.572, degrees of freedom = 11, probability level = 0.751, comparative fit index = 1.000, CMIN/DF = 0.688, root mean squared error of approximation = 0.000, and 90% CI = 0.000–0.029.

Our second multigroup SEM on the association between perceived racial discrimination and anxiety symptoms also showed a good fit (chi-square = 7.572, degrees of freedom = 11, probability level = 0.751, CFI = 1.000, CMIN/DF = 0.688, RMSEA = 0.000, 90% CI = 0.00–0.03). According to this model, change in perceived racial discrimination from 2002 to 2003 predicted change in anxiety symptoms from 2000 to 2012 in males (*B* = 0.23, *p* = 0.018) but not females (*B* = 0.09, *p* = 0.356). Gender differences were not found in the effect of age on perceived discrimination or anxiety symptoms; however, gender altered how parental employment and living in an intact family were predictive of baseline or changes in anxiety symptoms (Figure 2; Table 2).

**Table 2:** **Summary of path analysis between changes in racial discrimination and changes in anxiety symptoms among male and female African-American youth**.

	Men		Women	
	*B* (SE)	*P*	*B* (SE)	*P*
**Paths of interest**				
Racial discrimination 2003 → anxiety symptoms 2012	0.23 (0.00)	0.018	0.09 (0.00)	0.356
Racial discrimination 2000 → anxiety symptoms 2000	0.21 (0.00)	0.007	0.42 (0.00)	<0.001
**Auto-regressive paths**				
Racial discrimination 2000 → racial discrimination 2003	0.55 (0.05)	<0.001	0.43 (0.05)	<0.001
Anxiety symptoms 2000 → anxiety symptoms 2012	0.34 (0.09)	<0.001	0.28 (0.09)	<0.001
**Control paths**				
Age → anxiety symptoms 2000	0.11 (0.00)	<0.001	0.10 (0.00)	<0.001
Age → anxiety symptoms 2012	0.06 (0.01)	<0.001	0.06 (0.01)	<0.001
Age → racial discrimination 2003	0.02 (0.51)	0.001	0.01 (0.34)	0.002
Two parents working → anxiety symptoms 2000	−0.12 (0.08)	0.077	−0.05 (0.07)	0.442
Two parents working → anxiety symptoms 2012	−0.09 (0.10)	0.268	0.04 (0.10)	0.634
Two parents working → racial discrimination 2003	−0.03 (9.71)	0.651	−0.02 (6.52)	0.759
Intact family → anxiety symptoms 2000	−0.02 (0.09)	0.830	−0.01 (0.09)	0.829
Intact family → anxiety symptoms 2012	−0.05 (0.11)	0.517	−0.02 (0.13)	0.812
Intact family → racial discrimination 2003	0.05 (10.62)	0.529	0.21 (7.80)	0.002

## Discussion

Our study revealed major gender differences in the role that an increase in perceived racial discrimination during adolescence plays in deterioration of psychological well-being among Black youth in their transition to young adulthood. An increase in perceived racial discrimination was predictive of an increase in symptoms of anxiety and depression among Black males, but not Black females. Thus, perceived racial discrimination during adolescence has significant harmful effects on the mental health of Black men decades later. Although available literature has shown similar results for depression, this study is pioneer in showing that same pattern can be also observed for anxiety symptoms.

In a recent study among Arab Americans, perceived discrimination was only associated with psychological distress among males but not females (29). Other researchers have suggested that various types of stressors may have a larger effect on men than women (46–48). Assari et al. (46) found that an increase in neighborhood stress predicted a higher increase in depressive symptoms among male youth compared to their female counterparts. In another study, Assari and Caldwell (47) found that neighborhood stress predicted the risk of major depressive disorder (MDD) only in Black males but not Black females. Finally, in another 25-year cohort of Black and White adults, stress at baseline better predicted future risk of MDD in men than women (29). All these findings suggest that men may be more prone to the effect of various types of stressors including discrimination on risk of psychopathology.

It is argued that the mental health effects of perceived discrimination depend on demographic, social, and cognitive factors; however, most of this literature has exclusively studied depression and less is known for anxiety. Gender (29, 30, 49), socioeconomic status (18, 50), masculinity (51), racial identity (3, 52–54), and racial attribution (55) all alter the effects of discrimination on depression and psychological distress. Discrimination seems to be more harmful for men than women (29, 30), and even more deleterious for men who endorse higher masculine ideologies (51). Thus, being a man and also holding strong masculine attitudes and beliefs are vulnerability factors that increase harmful effects of discrimination.

Masculine role norms moderate the link between perceived racial discrimination and depressive symptoms in Black men (56). Higher restrictive emotionality is associated with more depressive symptoms in Black men of 20s and 30s, and higher self-reliance is associated with less depressive symptoms among them (56). Racial identity also plays a pivotal role in an individual’s encounters with racial discrimination (57). Overall, gender, context, and culture interact in shaping behaviors and well-being of Black adolescents (57).

Notably, researchers have found that Black men report higher rates of perceived discrimination compared to Black women (58, 59). Scholars have posited two hypotheses concerning the gender gap in perceived discrimination among Blacks. One is the subordinate male target hypothesis which argues that Black men are subject to more experiences of discrimination (32). Another view is the race–gender intersectionality hypothesis which argues that the gender-related measurement bias is the main culprit for the gender gap in perceived discrimination (32, 60). Ifatunji and Harnois used data from Detroit Area Study and the National Survey of American Life and found that the intersectionality hypothesis supported findings associated with major life discrimination, while the subordinate male hypothesis explained their findings related to everyday discrimination (32).

Most mental health researchers studying the consequences of perceived racial discrimination have focused on depression (3, 61) with few studying the effect of racial discrimination on anxiety. Considering race as the reason behind discrimination was a predictor of generalized anxiety disorder in African-Americans but not in non-Hispanic Whites (62). In another study, daily life experiences of discrimination rather than major experiences of discrimination were associated with social anxiety disorder (22). Perceived racial discrimination increases the likelihood of evaluating social interactions as harassing (28). Blacks who worry about interaction with other ethnic groups as the result of cultural mistrust are at high risk of social anxiety (63). Yet most of this research included adult samples and focused on depression as the outcome. Extending research on discrimination and mental health more generally to adolescents and young adults is a gap in our knowledge that requires attention.

Black males report higher rates of perceived discrimination compared to Black females (57–59, 64, 65). Concerning this gender gap two hypotheses have been proposed: race–gender intersectionality hypothesis which considers gender-related measurement bias as the main reason behind the mentioned gender gap and subordinate male hypothesis which considers more experiences of discrimination in Black men as the main cause of the gender gap in perceived discrimination (32). Researchers have suggested that findings on experiences of major life discrimination tend to be justified by intersectionality hypothesis, while experiences of everyday discrimination are better justified according to subordinate male hypothesis (32). Other researchers also found that everyday discrimination mediates the effect of incarceration history on discrimination for Black men (66).

Considering the subordinate male hypothesis, negative attitude toward Black males compared with the Black females has been reported as the result of stereotypes that exist around the intersection of race and gender including attribution of aggression and anti-intellectuality to Black males (57, 65, 67, 68). Black men have been stereotyped as “endangered, aggressive, angry, superhuman, subhuman, lazy, hyperactive, jailed, and paroled, on probation, lost, loveless, incorrigible, or just simply self-destructive” (69, 70). Police brutality and neighborhood crime and mass incarceration also effect Black men more than any other sociodemographic group (66). Black males receive relatively more negative treatment in schools compared to females (57, 71–75). Black boys are subject to more parental messages regarding racism and discrimination compared with the girls (76, 77), which in turn may increase the awareness of racism and vigilance for discriminatory cues. These notions may contribute to the differences in long-term consequences of perceived discrimination between Black males and females. A prospective longitudinal study on the effects of perceived racial discrimination in 14- to 21-year-old adolescents on health behaviors in 30s showed that Black men lean toward substance abuse while Black women tend to reduce exercise and physical activity (30). Also, discrimination showed a stronger cross-sectional association with smoking in men than women (31). These studies support our findings.

Researchers have also documented differences between males and females in coping processes subsequent to racial discrimination (57, 78). Black women have a higher tendency to use an avoidant coping mechanism in response to race-related stress as compared with Black men (78). This pattern is very different from men who may use more combative forms of coping such as using ones voice as power, or John Henryism (79). In a study among Blacks, for men, as John Henryism increased, blood pressure and the risk of hypertension increased. For women, however, as John Henryism increased, blood pressure and the risk of hypertension decreased (79).

Avoidant and confronting coping may mediate the association of gendered racism with heightened distress (78). Black women lean to their social support, including family and friends, as a coping strategy when they experience gendered racism (80–83), which makes women better users of social support. In line with this argument on importance of social support is the finding that positive relationships with sons are associated with less depressive symptoms and drinking behavior in Black fathers (51). Finally, Black men more commonly get discriminated against in the labor market, as Black women have better job opportunities (33). This may be in part due to stereotypes or public policies regarding judiciary system (e.g., mass incarceration and stop and frisk and brutal policing).

Our findings have implications for future research as well as public health and clinical practice, especially as it relates to Black men. Mental health practitioners may want to ask Black men with depression about their perceived racial discrimination over the life course to better understand their depression. Further research is warranted to scrutinize the mechanisms behind the heightened effects in Black men. Some potential suspects are masculinity, ineffective coping, vigilance, and racial attribution, or racial identity that may alter men’s response and vulnerability to discrimination. It may be especially useful if this line of research went beyond individual level variables to capture structural factors such as poverty, racism, police brutality, neighborhood disorder, and blocked opportunity.

This study is subject to a number of limitations. First, brief symptom inventory was used to measure anxiety and depression symptoms. Future research should use structured interviews that evaluate diagnostic criteria for psychiatric disorders. Second, our models did not account for antidepressants or anxiolytic medications which may mask the symptoms. Third, the study did not account for the role of gender discrimination in this study. Finally, we did not consider race-related identity and attribution of racism. Racial identity and a wide range of other factors such as school achievement, occupational situation, and SES could possibly moderate the same association, but were out of the scope of this study. Thus, it is still unknown whether it is gender *per se* or other confounders such as SES or occupation status that alter the effect of discrimination on mental health. Our argument that gender itself may alter the effect of discrimination is supported by studies showing that the effect of stress and discrimination is higher in the presence of high masculinity beliefs (51). Despite these limitations, our findings contribute to the available scientific literature on developmental effects of discrimination on mental health. Our 18-year cohort of several hundred Blacks provides compelling evidence of the long-term negative effects of discrimination. In addition, using SEM enabled us to test the associations between changes in the discrimination and outcomes over time which provides powerful support for the casual linkages studied especially given that we controlled for some potentially spurious variables.

In conclusion, the current study, as well as previous research, suggests significant gender differences in effects of perceived racial discrimination on Black’s symptoms of anxiety and depression. Black males are particularly vulnerable to the effects of an increase in perceived racial discrimination on their symptoms of anxiety and depression. Further investigation is needed to elucidate the most effective ways to prevent these effects as a salient determinant of psychopathology among Black males.

## Ethics Statement

The University of Michigan Institutional Review Board approved the study protocol for all years of data collection and all participants provided assent and consent for participation in the study.

## Author Contributions

SA developed the original idea of this analysis and also analyzed the data. EM-Z drafted the manuscript and revised the paper. MAZ and CHC contributed to design and data collection of the original study. MAZ and CHC contributed to interpretation of the findings and revision of the manuscript. All authors confirmed the final version of the manuscript.

## Conflict of Interest Statement

The authors declare that the research was conducted in the absence of any commercial or financial relationships that could be construed as a potential conflict of interest.
