# Supplementary material for: Racial Discrimination during Adolescence Predicts Mental Health Deterioration in Adulthood: Gender Differences among Blacks
Source: Front Public Health. 2017 May 29;5:104. doi: 10.3389/fpubh.2017.00104 (PMC5447045; doi:10.3389/fpubh.2017.00104)
Supplement: Supplementary file 1 [file data_sheet_1.docx]

**Appendix**

These questions ask you to think about experiences that some people have as they go about their daily lives. Please FIRST determine how often you have experienced each event because of your **RACE** in the last 12 months. Use the scale in the first column and circle the appropriate number. NEXT, use the scale in the second column to indicate how much it bothers you when the experience happens. Circle the appropriate number in each column.

**IN THE LAST 12 MONTHS**… How often because of your **RACE**? How much does it bother you?

0=never happened to me 0=never happened to me

1=once or twice 1=doesn't bother me at all

2=a few times 2=bothers me a little

3=about once a month 3=bothers me somewhat

4=a few times a month 4=bothers me a lot

5=once a week or more 5=bothers me extremely

1. Being ignored, overlooked, or not given service 0 1 2 3 4 5 0 1 2 3 4 5

(in a restaurant, store, etc.)

2 Being treated rudely or disrespectfully 0 1 2 3 4 5 0 1 2 3 4 5

3 Being accused of something or treated suspiciously 0 1 2 3 4 5 0 1 2 3 4 5

4 Others reacting to you as if they were afraid or

intimidated 0 1 2 3 4 5 0 1 2 3 4 5

5. Being observed or followed while in public places 0 1 2 3 4 5 0 1 2 3 4 5

6 Being treated as if you were "stupid", being "talked

down to" 0 1 2 3 4 5 0 1 2 3 4 5

7 Your ideas or opinions being minimized, ignored

or devalued 0 1 2 3 4 5 0 1 2 3 4 5

8 Overhearing or being told an offensive joke or

comment 0 1 2 3 4 5 0 1 2 3 4 5

9 Being insulted, called a name, or harassed 0 1 2 3 4 5 0 1 2 3 4 5

10 Others expecting your work to be inferior 0 1 2 3 4 5 0 1 2 3 4 5

11 Not being taken seriously 0 1 2 3 4 5 0 1 2 3 4 5

12 Being left out of conversations or activities 0 1 2 3 4 5 0 1 2 3 4 5

13 Being treated in an "overly" friendly or superficial way 0 1 2 3 4 5 0 1 2 3 4 5

14 Other people avoiding you 0 1 2 3 4 5 0 1 2 3 4 5

15 Being mistaken for someone who serves others

(i.e., janitor, bellboy, maid) 0 1 2 3 4 5 0 1 2 3 4 5

16 Being stared at by strangers 0 1 2 3 4 5 0 1 2 3 4 5

17 Being laughed at, made fun of, or taunted 0 1 2 3 4 5 0 1 2 3 4 5

18 Being mistaken for someone else of your same 0 1 2 3 4 5 0 1 2 3 4 5

race

19 Being unfairly stopped, searched, questioned,

physically threatened, or abused by the police 0 1 2 3 4 5 0 1 2 3 4 5

J10t. Not being hired for a job 0 1 2 3 4 5 0 1 2 3 4 5
